# Analysis of Food Security of Older Rural Indigenous People in Latin America and the Caribbean

**DOI:** 10.3390/foods13111772

**Published:** 2024-06-05

**Authors:** Angélica Hernández-Moreno, Olga Vásquez-Palma, Fernanda Gutiérrez-Gutiérrez, Otilia Cordero-Ahiman, Natalia Celedón-Celis, Jorge Hochstetter-Diez

**Affiliations:** 1 Departamento de Salud Pública, Facultad de Medicina, Universidad de La Frontera, Temuco 4780000, Chile; angelica.hernandez@ufrontera.cl (A.H.-M.); natalia.celedon@ufrontera.cl (N.C.-C.); 2 Centro de Estudios y Promoción de los Derechos Humanos, Universidad de La Frontera, Temuco 4780000, Chile; 3 Departamento de Procesos Terapéuticos, Universidad Católica de Temuco, Temuco 4780000, Chile; ovasquez@uct.cl; 4 Departamento de Ciencias de la Computación e Informática, Universidad de La Frontera, Temuco 4780000, Chile; fernanda.gutierrez@ufrontera.cl; 5 Departamento de Economía, Empresa y Desarrollo Sostenible, Universidad de Cuenca, Cuenca 010201, Ecuador; otilia.cordero@ucuenca.edu.ec

**Keywords:** food insecurity, older adults, Latin America and the Caribbean, indigenous contexts, rural areas

## Abstract

Food insecurity is a critical issue in the Americas, with severe impacts in the Caribbean, Mesoamerica, and South America, particularly affecting older adults in Indigenous and rural contexts where it intersects with poverty, gender, and ethnicity. This study aims to provide an in-depth understanding of the current research about food insecurity among older Indigenous adults in Latin America and the Caribbean. A comprehensive literature review was conducted, utilizing specific search queries and the population, intervention, comparison, and outcome (PICO) strategy across multiple databases to identify the pertinent studies. The findings indicate an increase in academic output on this topic since 2018, with significant emphasis on the interplay between climate change and food insecurity. The review highlights the importance of developing targeted food programs, reforming policies, and fostering collaboration between academia and local communities to implement practical interventions. Despite the growing body of literature, a notable research gap persists in rural areas of Latin America and the Caribbean. This study underscores the necessity of balancing the geographic distribution of research and emphasizes the preservation of cultural practices and the adaptation of public policies to support traditional food practices. It advocates for culturally sensitive interventions and interdisciplinary collaboration to formulate comprehensive strategies. The originality and value of this study lie in its focused analysis of older Indigenous adults, contributing crucial insights to the international literature on food security.

## 1. Introduction

The continued existence of significant inequalities in access to healthy and adequate food worldwide is a dehumanizing and immoral situation [1], it should, therefore, be an ethical priority to influence the governance systems of nations and international organizations to eliminate these inequalities.

Food insecurity affects 29.6% of the world’s population at moderate or severe levels, which implies a lack of access to adequate food [2]. Food insecurity is also concentrated among vulnerable groups such as women, children, rural populations, Indigenous Peoples, the elderly, and migrants [3]. In the moderate or severe condition, in 2022, food insecurity reached 33.3% of adults living in rural areas, compared to 28.8% in peri-urban areas and 26.0% in urban areas; this situation was more frequent in women, 27.8% of them compared to 25.4% of men [4]. Reduced availability in rural areas; higher food costs, with increases of 6.7% between 2019 and 2021; and a decline in incomes given the post-pandemic downturn in the economy have left consequences [2].

Currently, there exists a critical situation, a breaking point that impacts the capacity to maintain food production for a growing world population and poses serious problems of inequity in its distribution, particularly in regions of greater social and economic vulnerability [5]. The prevailing system of production, distribution, and consumption is the main cause of the environmental degradation that affects the health of ecosystems, and therefore, it is necessary to substantially modify it [6]. Among the sustainable production systems that are more resilient to climate change are smallholder and Indigenous Peoples’ agriculture, which presents marginal conditions of productivity and postponement due to the vertical approach of the global production system that excludes them. It is, therefore, vital to understand the food security situation of these groups [7]. This is addressed in this study, which is intended to contribute to the knowledge of food security of one of the most vulnerable groups, older rural Indigenous People in Latin America and the Caribbean (LAC).

With this sector having an important mitigating role in times of economic crisis and its impacts on the population [8], food security is a fundamental aspect of ensuring the well-being and quality of life of older Indigenous people in Latin America; and it is especially relevant for older Indigenous people living in rural areas, as they face additional challenges due to socio-economic, geographic, and ethnic factors. These factors can contribute to chronic malnutrition in this population, which can have negative impacts on their overall health and well-being [9], as well as intensify existing pathologies and add negative emotional factors that undermine mental health [10].

The dimensions of food security have been widely discussed by the Food and Agriculture Organization of the United Nations (FAO) and described as follows: availability, referring to the existence of sufficient food for consumption; food access, defined as the possibility of the population to obtain and choose adequate food; utilization or bioavailability, understood as how the body takes advantage of the nutrients consumed; and stability, comprehended as the permanence of the aforementioned dimensions [11].

In the LAC region, it has been observed that the trends in increasing food insecurity and overweight–obesity are related to populations in marginalized areas, women, farmers, Indigenous Peoples, and rural areas [2,12]. It is important to emphasize that while social programs have been implemented to address food security and reduce malnutrition, they have tended to focus primarily on combating child malnutrition and hunger, especially since the impacts of the 2008 food and financial crisis [13,14,15]. However, attention needs to be paid to the situation of older Indigenous people in rural areas, as they face specific challenges that require solutions adapted to their needs, beyond the perspective of poverty, considering cultures, life experiences, and revitalization towards sustainable food systems [16,17]. In this sense, the research problem is the existing gap in the production of the scientific literature on the topic in LAC territories, which can be associated with the presence of a gap in the current state of knowledge, which is addressed by this study through a systematic mapping that allows the location of studies on the topic.

The objective of this study is to analyze the existing initiatives in the literature on food security in older Indigenous Peoples in LAC and to contribute to the knowledge through an analysis of the food insecurity situation of these groups and to survey the production of knowledge and trends related to the subject of study.

This text is organized as follows: it begins with the background in Section 2. Section 3 discusses the relevant previous research. The methodology of the systematic mapping is detailed in Section 4. The results of the study are presented in Section 5. Finally, Section 6 shows the findings on initiatives or strategies related to food security. Finally, the conclusion and future work is presented.

## 2. Background

The concept of food security refers to the availability and economic, cultural, and permanent access to healthy, sufficient, and nutritionally adequate food to meet people’s needs [15]. When this is not achieved, there is food insecurity at some level. When access is uncertain, it is referred to as mild food insecurity; when quality, variety, and quantity are compromised, it signifies moderate food insecurity; and when individuals frequently experience food deprivation, possibly going a day or several days without eating, it indicates severe food insecurity [18].

The prevalence of moderate or severe food insecurity in the Americas is higher than the world average. The UN 2022 report on food and nutritional security in LAC states that 22.5% of its inhabitants are food insecure. The distribution of these deficiencies is unequal in the three regions that constitute it; in the Caribbean, the number is 52%; in Mesoamerica, it is 27%; and in South America, it is 18.4% [2].

Today’s production and food systems are very vulnerable to the conflicts occurring in the world, to environmental and financial crises, and even more so in vulnerable territories [4].

In addition, this situation is associated with the high cost of food, especially for healthy diets [2], and aggravated by the poor access of the elderly to these, due to low pensions. This is why the elderly are considered a vulnerable population group, due to the economic precariousness they face, which causes them food insecurity [19]. An elderly person is defined as a person 60 to 65 years of age or older, according to LAC national legislation [20]. It has been shown that the condition of elderly people associated with both Indigenous and rural contexts increases this insecurity [21,22,23].

Studies in several LAC countries have shown the correlation between food insecurity and poverty, gender, rurality, and ethnicity, which indicates the impact of structural problems affecting food access in these groups [3,24,25,26].

When public policies tend to solve structural, transversal, and intersectoral problems, they provide a solution to the situation raised, so it is not enough to develop a public policy related to food security for the elderly or rural Indigenous people separately, but it implies a commitment of the states and their institutions with an intersectional and comprehensive methodology to address the problem presented here. In addition, regional integration is relevant in solving the great inequality in access to healthy diets for the entire population.

## 3. Materials and Methods

**Figure 1:**
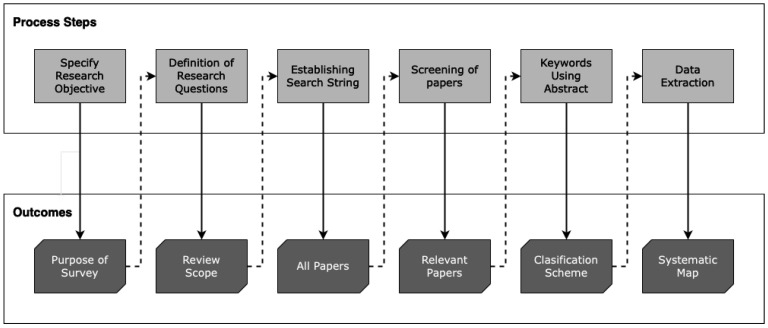
Stages of the systematic mapping process.

The methodology consists of following the procedures of the systematic mapping technique, which facilitates the identification, categorization, and analysis of the relevant literature for a specific research topic. It is a commonly used technique to systematically address one or more research questions. The steps that make up the adapted systematic mapping process are detailed in Figure 1.

### 3.1. Objectives and Research Questions

The objective of this systematic mapping is to locate articles involved in debates that analyze the existing initiatives on food security of older Indigenous people in LAC. Table 1 details the specific research questions that were formulated to gather relevant information and recognize trends related to the topic under study. From these questions, the aim is to attain a complete and thorough understanding of the subject, thereby approaching it in a more meaningful way.

### 3.2. Research Questions to Be Applied

Table 2 describes the search strings used to conduct an exhaustive search of the data sources, composed of study-specific keywords to answer the research questions. The search for articles was carried out using the population, intervention, comparison, and outcomes (PICO) strategy.

### 3.3. Data Extraction

To support the present study, a comprehensive data extraction search was performed, implementing a strategy based on the selection of the main sources of digital databases of scientific publications, such as Web of Science (WoS), Scopus, and EBSCO. The use of these sources allowed access to important studies of the relevant academic and scientific literature, collaborating and enhancing the knowledge base for this analysis. In addition, specific criteria were established for an optimal and precise search, paying particular attention to the study of titles and abstracts of documents. Choosing to use this functionality (available in all the databases consulted) was fundamental to simplify and facilitate the identification of the academic and scientific articles that were more relevant to achieving the objective of this research.

### 3.4. Inclusion and Exclusion Criteria

The articles selected from the data sources were then filtered according to the following inclusion and exclusion criteria.

#### 3.4.1. Inclusion Criteria

- Research articles in English from scientific journals and conferences.
- Articles that consider the food security of older rural Indigenous people in the world.
- Articles published since 2010.

#### 3.4.2. Exclusion Criteria

- Articles not in English.
- Articles before 2010.
- Articles that do not include topics related to food security of older rural Indigenous people.
- Duplicate studies in different databases.
- Incomplete items.
- Articles that are not in journals or conferences.
- Articles that are not available (open access).
- Items not relevant in the search string.
- Reviews (can be used in related works).
- Gray literature articles that are not in official government sources.

### 3.5. Search Execution

Table 3 shows the result of an initial cluster of 184 articles for the selected search string used in the chosen digital databases of scientific publications. The data collection was carried out using the export functions of each digital library.

### 3.6. Selection of Articles

**Figure 2:**
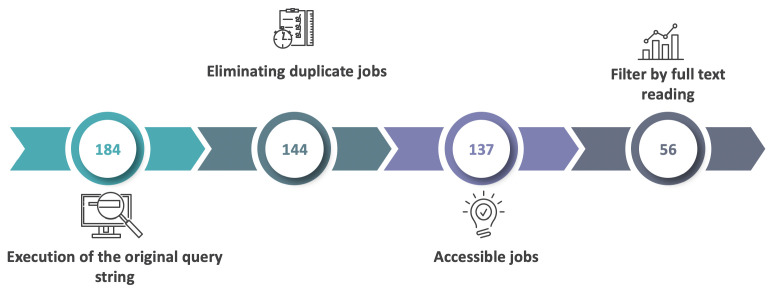
Search and selection process.

Figure 2 shows the articles’ search and selection process. After obtaining the initial assemblage of 184 articles, the duplicate articles were discarded, reducing the number to 144. The inclusion and exclusion criteria were then applied, reducing the number of articles to 137. The 137 articles were then read by two researchers separately (specifically, the abstract, introductions, and conclusions of each article), deciding which complied with the research purpose, and for those articles that were analyzed differently, the opinion of a third researcher was requested. From this process, 56 articles were selected to analyze, corroborate their relevance, and provide answers to the research questions.

### 3.7. Classification Scheme

The 56 selected articles (see Table A1 for a summary of the articles chosen mentioning food security of older Indigenous people in rural areas) were then categorized according to three dimensions: chronology, category, and type of article.

The chronology dimension categorizes the articles according to their year of publication, taking into account that articles published from 2010 onward were chosen.

The category dimension corresponds to the context in which the research was conducted, i.e., the following scenarios: child food and nutrition, climate change and environment, Indigenous culture and traditions, technological integration and community development, traditional agricultural and food practices, food sovereignty and security, diversity and ecosystems, food education and awareness, food insecurity, nutritional transition, and community health.

In terms of type dimension, articles were classified into:

- Analysis: work related to the analysis of food security in older rural Indigenous people.
- Implementation experience: work related to studying the implementation of existing proposals for food security in older rural Indigenous people.
- Proposals: work proposing programs, research centers, and solutions related to food security in older rural Indigenous people.

It is important to mention that the articles were classified in only one of the categories of each dimension, according to the prevalence of the subject matter addressed.

### 3.8. Map Construction

As a result of the systematic mapping process focused on the food security of older Indigenous people in rural areas, a map has been developed to simplify the representation and analysis of the data collected in this particular context. This map is presented below in Section 4, showing a visual and organized representation of the categories generated from the studies incorporated in this mapping.

## 4. Results

In this section, the findings obtained through the classification and detailed analysis of the chosen studies are shown, after a thorough review and evaluation based on the literature pertinent to this research. The results presented here provide a broad overview of the academic and scientific literature related to the field of study.

### 4.1. Overall Analysis by Characteristics

This subsection presents the results obtained from the categorization and detailed analysis of the selected studies.

**Figure 3:**
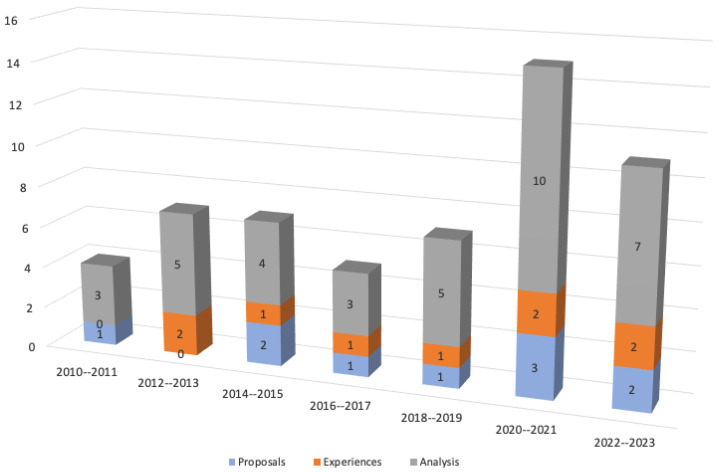
Distribution of the selected articles according to their years of publication.

Regarding the chronology dimension, Figure 3 shows the distribution of the studies according to their publication year range. Overall, it is explicit that there was a relatively constant volume of research in this field until 2018–2019 with an average of 7 publications per period, which then increased sharply in the period 2020–2021 with 15 publications, with a small drop in 2022–2023 (11 publications) but still showing an overall growth compared to the previous periods.

It is observed that the proportion of studies oriented to reviewing and analyzing literature about food security in older rural Indigenous people consistently comprise most of the work in this field throughout the examined years, while studies involving the exposition of experiences and the development and implementation proposals regularly account for approximately only up to one third of the total research production in this field.

**Figure 4:**
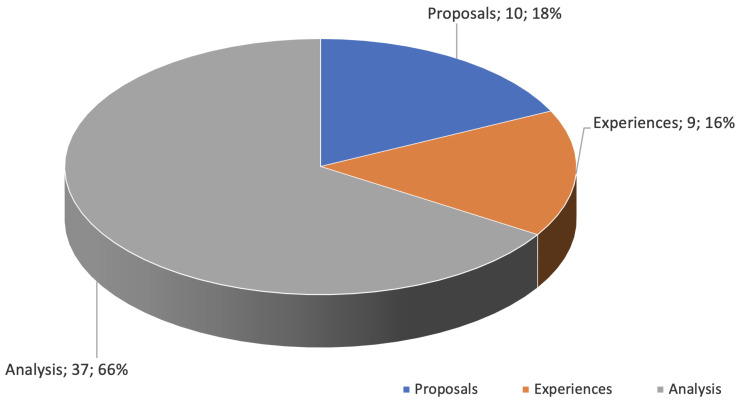
Classification of selected articles according to type dimension.

The dimension of the type of article provides an approach to answer the research questions concerning the proportion of articles that contribute to the topic of study, discovering those that focus explicitly on proposals or implementations of solutions for food security in older rural Indigenous people. Figure 4 represents the classification of the selected articles into three dimensions: analyses, experiences, and proposals. This was carried out by considering the following:

- The analysis focuses on reviewing relevant studies on food security in older Indigenous people residing in rural areas. This review aims to understand the particularities faced by these communities in terms of access to and availability of nutritious food, considering the effects of traditional agricultural practices, public policies, and the availability of local resources.
- Implementation experiences refers to studies that have investigated how food security proposals targeting older Indigenous people in rural areas have been put into practice. This review seeks to analyze the results, challenges, and lessons learned from the implementation of such strategies to better understand what factors contribute to the success or failure of these initiatives.
- The proposals reviewed in the literature encompass programs, the creation of research centers, and innovative solutions focused on improving the food security of older Indigenous people in rural areas. This analysis highlights initiatives that seek to address the specific challenges faced by this population, from the development of public policies to the implementation of community projects that promote sustainable agricultural practices and access to nutritious food. Special emphasis is placed on the relevance of adapting these solutions to the particular cultural and socioeconomic conditions of Indigenous communities to facilitate their acceptance and effectiveness.

From Figure 4, it can be observed that the majority of the articles selected (66% of the total) are focused on analyzing, describing, discussing, and/or comparing the literature on food security in older rural Indigenous people. Sixteen percent of the articles focus on studying the implementations of existing proposals for food security in older rural Indigenous people. Only 18% of the studies selected propose programs, research centers, or solutions related to food security in older rural Indigenous people.

**Figure 5:**
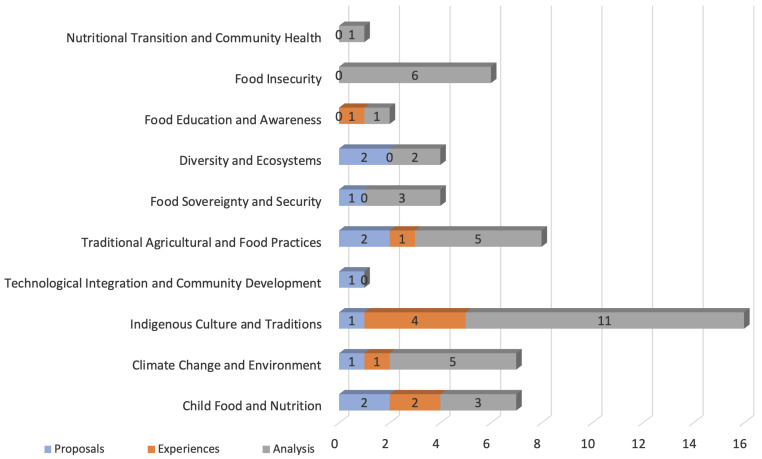
Distribution of selected articles according to category dimension.

The category dimension refers to the context in which the research was conducted. Figure 5 shows how the articles were classified and grouped into 10 contexts: child food and nutrition, climate change and environment, Indigenous culture and traditions, technological integration and community development, traditional agricultural and food practices, food sovereignty and security, diversity and ecosystems, food education and awareness, food insecurity, nutritional transition, and community health.

It should be noted that 16 papers focus on Indigenous Culture and Traditions, representing 28.57% of the total of articles, but only one is a proposed solution and the other 15 are distributed between analyses of studies and experiences from other papers. Similar situations can be observed in other contexts, such as climate change and environment and traditional agricultural and food practices, where the majority of the articles found for each one of them correspond to analyses. Moreover, contexts like national transition and community health and food insecurity are only addressed through analysis research, but they do not present any articles that address these issues through the implementation of experiences or the proposal of solutions.

### 4.2. Systematic Mapping

**Figure 6:**
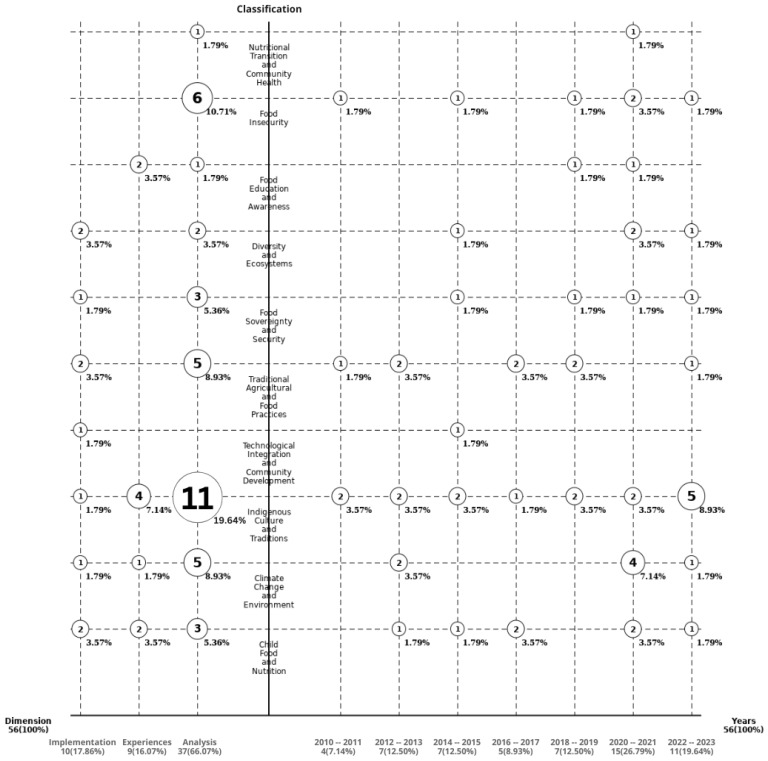
Selected studies arranged according to time of publication, type of article, and context.

A map, which is shown in Figure 6, was designed to illustrate and evaluate the selected works and presents them arranged in the three dimensions. For the chronological dimension, i.e., on the right side of the figure, the publications are classified by year ranges. On the left side, documents are grouped by article type. In the middle column, the categories of the study approaches are presented.

The map provides an overview of the related work on food security in older rural Indigenous people and facilitates visualizing three dimensions classified by time, category, and article type at the same time. Furthermore, it provides the opportunity to address the research questions, which are explored in the subsequent subsections.

### 4.3. RQ1: How Much Scientific Evidence Exists Regarding the Food Security of Older Indigenous People?

**Figure 7:**
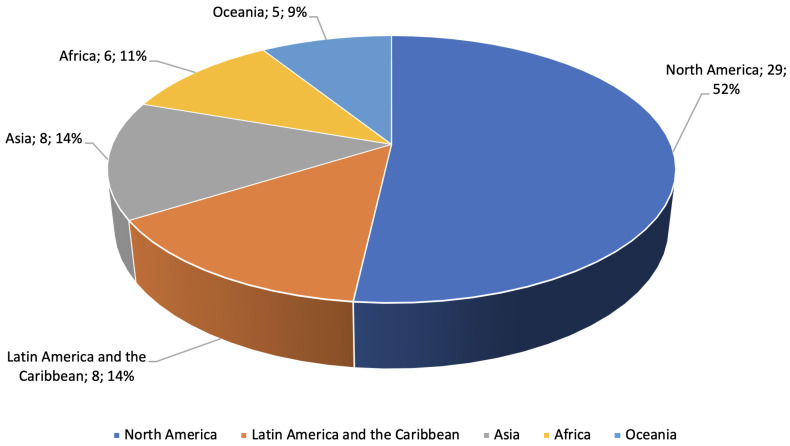
Classification of selected studies according to geographical location.

Figure 7 illustrates the classification of the selected studies according to the geographical location on which they are based, allowing further examination of the distribution and concentration of research production in the field.

Of the 56 papers found that relate to food security in older Indigenous people, the vast majority, 29 (52%), were conducted in North America, 79% of these in Canada; 8 of the total (14%) were conducted in LAC; 8 (14%) in Asia; 6 (11%) in Africa; and 5 (9%) in Oceania, all in Australia.

Of this total, 10 (18%) are implementation proposals, 37 (66%) are diet and food security analyses, and 9 (16%) are implementation experiences.

Of this universe, 31 studies that consider older Indigenous people as part of this universe were identified, either specifically or as part of a broader cross-cutting population that includes them; however, only 12 of these are specifically targeted at older Indigenous people.

These 12 papers focus on a variety of topics, most of them looking at the impact of climate change on food security, using qualitative methodology through interviews and, in one case, focus groups. Two papers address food insecurity based on secondary analysis of national surveys. Two papers look at the preservation of traditional knowledge and practices. The other papers look at a variety of issues such as the historical and cultural impact of food security, the influence of technical assistance programs on traditional food practices, and Indigenous markets. All of them use qualitative methodologies, and one of them also uses photovoice workshops. Only one study, which measures the social acceptability of traditional foods, uses a quantitative methodology through surveys and taste tests of preparations with traditional plants.

Of these 12 studies, 7 address the main issue of food security in older rural Indigenous people, 3 of which were carried out in LAC and included older people in the study as part of the general population or as cultural informants, but they were not aimed at addressing the specific problems of this age group, except for one study that was aimed at analyzing the food insecurity of older Indigenous and non-Indigenous people.

### 4.4. RQ2: Of the Articles Found, How Many Correspond to Works Related to Rural Areas of Latin America and the Caribbean?

Of the 56 papers related to the food security of older Indigenous people, 8 papers focused on rural areas in Latin America and the Caribbean. Of these, 6 studies, whose goals and findings are briefly described in Table 4, were related to older Indigenous people in LAC, and 4 of them specifically addressed rural populations. These studies were oriented towards the analysis of traditional foods, cultivation systems, and their incorporation into diets. The other two studies included national databases that involved both Indigenous and non-Indigenous and rural and urban populations.

### 4.5. RQ3: From the Articles Found, What Type of Initiatives Exist Regarding Food Security for Older Indigenous People?

In reviewing the articles related to the food security of Indigenous elders in LAC, six studies analyze the relationship between the conservation of traditional practices of cultivation and production of cultural foods by Indigenous Peoples’ communities with the presence of healthy diets, food insecurity or food security and food sovereignty. Two of the papers are the product of national survey analyses, Mexico and Brazil; while two studies are of a mixed approach, Ecuador and Chile; one of a qualitative approach, Ecuador; and one uses a comparative study of food sovereignty of the Indigenous Peoples in New Zealand and Peru.

The studies address the community in general as informants and include older people as such, but only two of them consider them key informants in a primary way. The results suggest that the preservation and promotion of traditional cultural practices of Indigenous Peoples for the production of healthy food have a favorable impact on the presence of healthy diets in the communities and are associated with agroecological balance. The comparative study concludes that Indigenous cultural principles correspond with concepts of food sovereignty, but not with dominant approaches to food security. The study conducted in Brazil (2023), whose data come from the analysis of the 2017–2018 national household budget survey addressing social inequalities and food insecurity in households headed by older people, in which 25 per cent self-declared themselves as Indigenous, found that 10.1% had a moderate to severe range of food insecurity. The study carried out in Chile (2023), which uses an ethnographic questionnaire aimed at people between 18 and 87 years of age from the Atacameño (100) and Pehuenche (90) Indigenous Peoples, concludes that the population hardly preserves its traditional eating habits, and that a westernized diet with a predominance of processed foods prevails. It also associates western cultural assimilation in the diet with an increase in the presence of non-communicable diseases and risk factors for the development of these.

Six studies targeted Indigenous populations in LAC, of which only two proposed solutions. One of them proposed targeting food programs to the most nutritionally deprived and vulnerable populations, and the other study proposed reformulating policies and programs to address food insecurity in older people, better meeting their needs. The other studies presented the issues and related evidence, either as evidence of good practice, but without proposing concrete solutions.

One of them, “Evolution of food aid programmes in Mexico through information from the ENSANUT-MC 2016”, whose objective was to evaluate the coverage of food aid programs (FAP) in Mexico implemented in the population of all ages. It was observed that 44% of households have access to some FAP, showing high coverage of food aid programs in Indigenous and low socio-economic households and with moderate or severe food insecurity. Concerning the older adults program, linked to 22.4% of households, evenly distributed in all socio-economic levels of the population although significantly higher in rural localities and the south region, it increased its coverage by 9.8% in the study period, given its social and nutritional vulnerability and the increase in this population. The study proposed the targeting of food programs in general in the most deprived and nutritionally vulnerable populations.

The results of the other study, “Food insecurity and social inequalities in households headed by older people in Brazil: a secondary cross-sectional analysis of a national study”, showed the magnitude of the levels of food insecurity in households headed by older people (29.1%), even though a lower prevalence is observed for the Brazilian population in general. This is given the important contribution of the stable income of the elderly, such as pensions, which, in 2003, constituted 75% of household income, making it a protective factor. The situation of moderate/severe food insecurity in households headed by elderly people is related to the perpetuation of historical trends in Brazil, associated with inequalities in income distribution and access to education, with greater disparity towards women and the Black and Indigenous populations. The study emphasizes the need for studies that integrate the areas of nutrition, public health, and gerontology and to reformulate and develop policies and programs aimed at addressing food insecurity among older people, better meeting their needs and giving them visibility to guarantee their basic human rights.

The remaining studies presented the issues and related evidence, or as evidence of good practice, but without proposing concrete solutions.

## 5. Discussion

This comprehensive review and systematic mapping of the literature aimed to identify articles that examine current initiatives related to the food security of older Indigenous people in rural areas of Latin America and the Caribbean.

Since 2018, there has been a notable increase in the production of food security studies of older Indigenous people, which can be attributed to several factors, including changes in government policies, a growth in public awareness of food insecurity among older Indigenous people, as well as impetus from non-governmental organizations [32,33]. This increase reflects a growing recognition of the importance of addressing the specific challenges faced by this demographic group.

It is possible to appreciate in these studies a shift towards a more practical and implementation-oriented approach. While analytical research is essential to understanding the magnitude of the problem, the implementation of practical and measurable solutions is crucial to making a significant impact on the food security of Indigenous elders. This transition may also signal greater collaboration between academia and the affected communities.

The geographic concentration of studies in North America, especially Canada, underscores the need to balance this scientific output to address the particular problems of LAC in a relevant way. Given the cultural diversity and differences in food systems, the research in these areas can respond to unique challenges and allow for the formulation of more targeted and culturally sensitive interventions. LAC present a disparity in food insecurity figures, highlighting the urgency of addressing food insecurity in these regions, especially in rural areas.

While the predominance of analytical studies highlights the importance of understanding the existing challenges, it also highlights the need to translate this knowledge into interventions applied to territories, communities, and people. The studies exploring the positive connection between the preservation of cultural practices and food security underline the importance of considering cultural context and Indigenous traditions when designing interventions. Adapting policies and programs to incorporate cultural elements can improve the acceptance and effectiveness of initiatives and decrease resistance to future interventions while respecting the identity and customs of Indigenous communities. In addition, the analysis of Western cultural assimilation in diet and its association with non-communicable diseases highlights a critical challenge in rural areas of LAC. This finding suggests the need for interventions that address food trends and promote their sustainability, in this sense, opportunities to strengthen food security through the revitalization of traditional food practices are observed.

Based on these findings, it is suggested that the research be expanded in rural areas of LAC, focusing on interventions that incorporate the preservation of cultural practices and promote food security. It also highlights the need for interdisciplinary collaboration between nutritionists, public health experts, anthropologists, and gerontologists to develop comprehensive and contextualized strategies. This discussion reflects the complexity of the issue and highlights the importance of addressing the food security of older Indigenous people from a cross-sectoral and culturally relevant perspective. Implementing concrete solutions and focusing on underrepresented geographic areas are crucial for moving towards a significant improvement in the quality of life for this demographic group, their communities, and the population at large, given the current climatic situation.

### Research Gaps

Based on the literature review, we can observe the existence of some gaps in the current research on food security among older Indigenous people in rural contexts in Latin America and the Caribbean. The most obvious of them lies in the low scientific productivity of the LAC territory concerning the subject matter analyzed, as most of the studies analyzed were focused on Canada and the United States. It would be important to expand the territorial sample in order to reach generalizable theoretical conclusions that would provide a comparative panorama between territories in the different regions.

As indicated in most of the studies included in this systematic review, joint community involvement is suggested, including different groups of interest (local Indigenous communities, academia, and governmental institutions) in order to obtain a holistic view of the problem and possible solutions. However, there is still a large gap in the development and application of such measures, and as mentioned above, ultimately, the result is a lack of experiences that can serve as examples for the generalization of theoretical and practical conclusions within the research for the creation and establishment of sustainable policies. Furthermore, hand in hand with the aforementioned, the implementation of Indigenous ancestral knowledge and traditional practices to address problems of malnutrition and safe access to food within Indigenous communities is cross-cutting in application studies. Nonetheless, the next step for further research in this area should be to generate proposals based on the feedback obtained through experience in the application of these practices, which could serve as a guideline for communities to develop appropriate tools.

Meanwhile, the link between food insecurity and the loss of Indigenous knowledge and culture is often addressed. The future research and the development of policy proposals should also be oriented towards the self-empowerment of Indigenous communities and enhancing the sense of community and the preservation of their cultural identity, which was lost due to the effects of colonization. Education on this matter is key to dealing effectively with food insecurity in a comprehensive manner.

## 6. Conclusions

Food security in the elderly population has been shown to represent a major public health challenge, with significant health and nutritional consequences [32,33,34,35]. This problem is influenced by a complex interplay of factors related to food access, which requires a comprehensive approach to address it [34], including individual characteristics and interpersonal aspects such as social capital and financial support [33,35]. Aging-specific vulnerabilities, such as decreased functionality and mobility, high prevalence of chronic diseases, and risk of social isolation, increase the risk of food insecurity [35]. In addition, older adults face particular financial challenges due to reduced productivity and dependence on fixed incomes, which increase the vulnerability of those without pensions [33]. On the other hand, considering the evidence presented, the government has a crucial role to play in creating and promoting policies to ensure food security for older adults, particularly in rural Indigenous communities. It is essential to prioritize policies that increase financial resources and social capital and improve physical environments for this demographic group. Although solutions may vary culturally and geographically, implementing measures to assess and monitor food insecurity in this segment of the population is critical [32,33,34].

This study expands the understanding of the research on food security issues faced by older Indigenous populations, highlighting the unique challenges and needs of this demographic and finding common ground to develop an answer to the constraints in facing this issue. It also provides a comprehensive categorization of the existing research, offering a structured overview that can guide future studies. In addition, it is remarkable the emphasis on Indigenous traditional practices and ancestral knowledge and their role in food security, which aligns with the broader theories of cultural sustainability and food sovereignty.

Given the scarcity of the research on the food security of older Indigenous people in rural areas of Latin America and the Caribbean, it is important to note that the results obtained so far are exploratory. In addition, it has become evident that the production of knowledge on this topic has focused mainly on the United States of America and Canada, which highlights the need to expand the research in other territorial contexts. More specifically, this research has shown a clear need to extend the research in Latin America and the Caribbean, especially in the generation of culturally relevant knowledge to address unique challenges and opportunities in the region.

This study has also uncovered the criticality of promoting food security initiatives that consider local Indigenous traditions, agricultural practices, and knowledge to develop more targeted and effective interventions. It also highlights the importance of promoting food sovereignty and sustainable agricultural practices to improve long-term food security, and it is also crucial to generate knowledge on the food security of specific groups such as the elderly, Indigenous, and rural people, given their relevance in the current context. These findings highlight the need to guide the future research and development interventions, as well as the formulation of relevant, efficient, and culturally sensitive public policies to address food security at the individual and community levels.

To effectively address food security challenges in vulnerable populations, this study has also pointed out that fostering cross-sectoral and community collaboration is crucial. A collaborative and interdisciplinary approach between academics, Indigenous communities, governments and non-governmental organizations can facilitate knowledge sharing and the implementation of practical solutions. Raising awareness of the food security challenges faced by older Indigenous people is critical, and communication and education efforts can help mobilize resources to address these fundamental issues. In addition, the future research should explore the impact of climate change on the food security of these communities.

Similarly, this study suggests the necessity of educational programs that not only raise awareness about the importance of Indigenous traditional food practices and their health benefits but also promote the integration of Indigenous knowledge into more comprehensive educational curricula to foster understanding and respect for these practices.

On the same line, it is worth discussing the relevance of this line of investigation as it holds a close alignment with Sustainable Development Goals (SDGs) 2 (Zero Hunger), 3 (Good Health and Well-being), and 11 (Sustainable Cities and Communities) by addressing food security and promoting sustainable agricultural practices, through its focus on the nutritional aspects of food security, and by emphasizing the preservation of Indigenous culture. Moreover, the study reinforces principles outlined in the United Nations Declaration on the Rights of Indigenous Peoples (UNDRIP), particularly regarding the right to maintain and strengthen Indigenous traditional food systems, highlighting the importance of including older Indigenous populations in discussions and the elaboration of policies related to food security, ensuring their voices and needs are addressed.

## Figures and Tables

**Table 1:** Research questions to be applied.

Research Question	Motivation
RQ1: How much scientific evidence exists regarding the food security of older Indigenous people?	Obtain information on works related to food security for older Indigenous people.
RQ2: Of the articles found, how many correspond to works related to rural areas of Latin America and the Caribbean?	To classify the works found in order to analyze those that propose solutions in terms of food security for rural older Indigenous people in Latin America and the Caribbean.
RQ3: From the articles found, what type of initiatives exist regarding food security for older Indigenous people?	To classify the works found in order to analyze those that propose solutions in terms of food security for older Indigenous people in rural areas in Latin America and the Caribbean.

**Table 2:** Search String composition.

Main concepts	“Food Security”, “Food Insecurity”, “Food Safety”, ”Food Deficit”, “Food Deficiency”, “Food Poverty”, “Elderly People”, “Elders”, “Older People”, “Older Adult”, “Old Age”, “Aged”, “Ageing”, “Indigenous”, “Indigenous People”, “Indigenous Population”, “Aboriginal”, “Indigenous Person”
Groups of terms	(“Food Security” OR “Food Insecurity” OR “Food Safety” OR “Food Deficit” OR “Food Deficiency” OR “Food Poverty”) (“Elderly People“ OR “Elders” OR “Older People” OR “Older Adult” OR “Old Age” OR “Aged” OR “Ageing”) (“Indigenous” OR “Indigenous People” OR “Indigenous Population” OR “Aboriginal” OR “Indigenous Person”)
Search string	(“Food Security” OR “Food Insecurity” OR “Food Safety” OR “Food Deficit” OR “Food Deficiency” OR “Food Poverty”) AND (“Elderly People“ OR “Elders” OR “Older People” OR “Older Adult” OR “Old Age” OR “Aged” OR “Ageing”) AND (“Indigenous” OR “Indigenous People” OR “Indigenous Population” OR “Aboriginal” OR “Indigenous Person”)

**Table 3:** Search result classified by data source.

Data Source	Abstract Selection
Web of Science	65
Scopus	92
EBSCO	27
Total	184

**Table 4:** Studies linking older rural Indigenous people in LAC.

Ref.	Goals	Findings
[27]	To evaluate the social, cultural, and nutritional potential of the intervention of a mothers’ club that promoted two indigenous vegetables (nettle/*Urtica dioica* L. and cow’s tongue/*Rumex obtusifolius* L.) in children’s diets.	Cultural, local foods reveal a high social, cultural, and nutritional contribution. It was estimated that vegetables have an important contribution of vitamins and oligo-elements to the recommended dietary intakes for children, and their use increased the food security of the community. Socially, mothers’ cooking clubs were encouraged, which promoted the self-efficacy and cultural identity of Quichua women.
[28]	To assess factors associated with change in food insecurity (FI) with the 2012 and 2018–2019 National Health and Nutrition Surveys (Ensanut).	Food insecurity (FI) in Mexico decreased by 4.8 percentage points in the period studied but persists as a severe public health problem in one in five of the most deprived households. The program beneficiaries had higher FI than non-beneficiaries. Households with adequate cooking facilities were more food secure.
[29]	To analyze the prevalence and factors associated with moderate/severe food insecurity in elderly-headed households.	The sample corresponds to 16,314 households headed by older people, 10.1% of whom were moderately/severely food insecure. Of these, 11.9% were women; 25.5% were self-declared Indigenous; 18.3% had no schooling; and 29.6% had a per capita income of up to half the minimum wage. Statistically significant factors were color/race, region, schooling, per capita household income, and social benefits received at home.
[17]	Examine the respective “principio del buen vivir” of Allin Kawsay/Buen Vivir in Peru and Mauri Ora in Aotearoa in safeguarding food security.	Indigenous food security policies associated with cultural and environmental indicators of well-being are consistent with conceptualizations of food sovereignty, which revitalize and contribute to an alternative sustainable food system, but not with dominant approaches to food security.
[30]	To explore, through the Indigenous community of Caliata in the Ecuadorian highlands, the factors that support or hinder sustainable Andean food systems.	Contributing factors are cultural, including the agroecological space defined by the territorial management of Pachamama, the Andean cosmovision, and the ecocentric approach to food sovereignty. Community dietary patterns are consistent with cultural principles, all of which reinforce sustainable Andean food systems that contribute to a lower burden of disease. Aspects such as population aging and gender-differentiated agriculture are demographic challenges affecting these systems.
[31]	To characterize risk factors for non-communicable diseases in two Indigenous Chilean populations with different environments and food livelihood strategies.	Few differences in dietary and physical activity patterns in both populations, as well as in their epidemiological risk factors, due to their cultural dietary assimilation with the general population.

## Data Availability

The original contributions presented in the study are included in the article, further inquiries can be directed to the corresponding author.

## Appendix A

**Table A1:** Articles mentioning food security of older Indigenous people in rural areas.

Number	Article	Cite	Brief Description
1	Poor nutrition from first foods: A cross-sectional study of complementary feeding of infants and young children in six remote Aboriginal communities across northern Australia	[36]	The study assesses the first foods of Aboriginal and Torres Strait Islander infants recruited in a nutrition program. Findings highlight challenges in dietary diversity and promote breastfeeding and nutrient-rich complementary foods to improve family food security.
2	Mixed-methods study identifies key strategies for improving infant and young child feeding practices in a highly stunted rural Indigenous population in Guatemala	[37]	Study shows limited awareness about child malnutrition in Guatemala’s rural Indigenous population, along with poor diversity diets and inadequate meal frequency. Fortified foods are costly. Community-based initiatives like education, engaging landowners in domestic food consumption discussions, and redirecting funds to fortified foods are suggested.
3	A Influence of micronization (infrared treatment) on the protein and functional quality of a ready-to-eat sorghum–cowpea African porridge for young child-feeding	[38]	Micronized and extrusion cooked sorghum and cowpea based porridge offers a nutritious meal for young children in Africa with excellent hydration properties and protein content, addressing nutritional needs and potentially enhancing food security in both urban and rural communities.
4	Adequate dietary intake and consumption of Indigenous fermented products are associated with improved nutrition status among children aged 6–23 months in Zambia	[39]	The study examined food consumption patterns of infants in Zambia, especially of fermented products and their association with nutrition and illnesses. It is shown that the frequent dietary intake of traditional fermented foods and child’s nutritional status are positively linked. It could be considered as an indicator of the non-optimized potential of indigenous foods’ impact on nutrition and health.
5	Ode’imin giizis: Proposing and piloting gardening as an indigenous childhood health intervention	[40]	The research examined the feasibility of reducing obesity among Indigenous families at risk for homelessness using gardening health intervention in a Northern Midwestern urban area. The initiative addressed food insecurity and access and enhanced healthy food awareness and ancestral knowledge through culturally appropriate interventions.
6	Development of an acceptable indigenous food diet for Pedi children under five years in early childhood development centers in rural Limpopo, South Africa	[41]	The study promotes indigenous food (IF) in early childhood development (ECD) centers for children’s nutrition, aligning with UN Sustainable Development Goal 2. Scaling up the use of an IF diet is suggested to preserve Indigenous knowledge and fulfill dietary requirements for children.
7	Use of traditional environmental knowledge to assess the impact of climate change on subsistence fishing in the James Bay Region of Northern Ontario, Canada	[42]	The study using traditional environmental knowledge (TEK) coupled with climate data revealed a relationship between climate change and the decreased number of fish through mortality and population dynamics, thus challenging food security for Canadian Aboriginals in remote areas.
8	Being on land and sea in troubled times: Climate change and food sovereignty in Nunavut	[43]	Results show climate change heightens the existing socio-economic factors, thus eroding food sovereignty in the Canadian Arctic. A communal approach is crucial for effective food insecurity mitigation efforts.
9	Assessment of exposure to perfluorinated industrial substances and risk of immune suppression in Greenland and its global context: a mixed-methods study	[44]	The study reveals high PFAS exposure in Inuit from consuming marine mammals, posing substantial health risks. It states the importance of mapping and evaluating the exposure in countries involved.
10	“We Hardly Have Any Moose Around Here Anymore”: Climate Change and the Barriers to Food Security in the Dehcho Region, Northwest Territories	[45]	Climate change and socio-economic factors impact food security in rural Indigenous communities in northern Canada. Challenges include changing ecosystems, weather unpredictability, non-local harvesters, loss of traditional knowledge, and high living costs. The research informs policies for understanding local needs in subarctic regions.
11	Linkages between human health and ocean health: A participatory climate change vulnerability assessment for marine mammal harvesters	[46]	Climate change and industrial development threaten Indigenous marine mammal hunting in Alaska’s Bering Strait region. Key concerns are noise, pollution, and food source depletion. Recommendations are incorporating local knowledge for adaptation initiatives and urging funding agencies to prioritize practical tribal research.
12	The Food Equity and Environmental Data Sovereignty (FEEDS) Project: Protocol for a Quasi-Experimental Study Evaluating a Digital Platform for Climate Change Preparedness	[47]	The Food Equity and Environmental Data Sovereignty (FEEDS) Project employs citizen science for real-time climate change impact monitoring on food sovereignty and mental well-being, promoting Indigenous self-determination and governance and providing communities with appropriate tools to respond to crises opportunely.
13	Community-based Participatory Process—Climate Change and Health Adaptation Program for Northern First Nations and Inuit in Canada	[48]	Health Canada’s program empowers northern communities for community-based research on climate change impacts, fostering adaptation plans and information dissemination, increasing their understanding of climate change impact on health and developing local strategies.
14	The co-production of knowledge reveals the loss of Indigenous hunting opportunities in the face of accelerating Arctic climate change	[49]	Reduced sea ice in Alaska’s northwest affects ugruk (bearded seal) hunting, impacting Indigenous communities’ food security, well-being, and culture.
15	Understanding perceptions of food access issues and responding initiatives in Fort Providence, NWT	[50]	Remote Indigenous communities in Northern Canada face challenges in maintaining food security due to socio-cultural, economic, and environmental barriers. Local, community-driven, land-based food procurement programs contribute to education, cultural continuity, community, and skill building. The programs’ dependency on funding and human capacity makes them vulnerable to external factors.
16	Associations between household food insecurity and health outcomes in the Aboriginal population (excluding reserves)	[51]	Aboriginal adults facing food insecurity are more likely to present poor general and mental health, less sense of community and high stress, compared with food-secure households.
17	Conceptualization, experiences, and suggestions for the improvement of food security among Aboriginal and Torres Strait Islander parents and carers in remote Australian communities	[52]	The study shows perceptions of food-insecurity in remote Aboriginal communities. Factors contributing to food insecurity as well as coping mechanisms are detailed. Improving transport access, income stability, and electricity support is crucial for lasting solutions.
18	Adaptation of Indigenous communities in kampung cireundeu to the environment in maintaining local wisdom	[53]	Cireundeu community adapts by planting cassava due to unsuitable conditions for rice. Efforts focus on maintaining local wisdom for food security.
19	Exploring First Nation Elder Women’s Relationships with Food from Social, Ecological, and Historical Perspectives	[54]	Indigenous women, disproportionately affected by colonization, face health disparities linked to residential school intergenerational trauma and cultural loss, impacting food practices and food security.
20	Community food program use in Inuvik, Northwest Territories	[55]	Individuals chronically supported by community food programs (CFPs) in the Canadian Arctic are more likely to be women, Aboriginal, housing insecure, unemployed, and educationally disadvantaged, highlighting the need for policy attention.
21	Inuit culture and opportunity recognition for commercial caribou harvests in the bio-economy	[56]	Inuit culture guides caribou use in the northern bio-economy, emphasizing community decision-making, use of traditional resources, food security, and benefits for health and economic development.
22	Registered Indian children’s school success and intergenerational effects of residential schooling in Canada	[57]	Off-reserve registered Indian children’s school success is influenced by factors like household income, gender, food security, and parental residential school attendance.
23	Fishing methods, catch composition and exploited biomass in the Aby lagoon (Adiaké, Côte d’Ivoire)	[58]	The effective management of Ivorian lagoon water bodies is concerning, principal limitations are individuals non-submission to fishery administration, struggle for space, and use of non-selective gears. Sustainable development training and adherence to management rules are recommended for rational exploitation.
24	Linking health and the environment through education—A Traditional Food Program in Inuvik, Western Canadian Arctic	[59]	The traditional food program in Inuvik engaged students in the full cycle of traditional food procurement, enhancing skills and promoting long-term food security, language learning, and community connections.
25	Examining Pathways from Food Insecurity to Safer Sex Efficacy Among Northern and Indigenous Adolescents in the Northwest Territories, Canada	[60]	Food insecurity in Northwest Territories adolescents indirectly affects their sexual well-being, emphasizing the need for holistic interventions addressing sexual and mental health in the context of poverty.
26	Impact of Assistance Programs on Indigenous Ways of Life in 12 Rural Remote Western Alaska Native Communities: Elder Perspectives Shared in Formative Work for the “Got Neqpiaq?” Project	[61]	Alaska Native Elders express concerns about the impact of government assistance on Indigenous culture, calling for considerations in meeting food security and education needs without disrupting traditional practices.
27	The use of traditional climate knowledge by the Iraya Mangyans of Paluan, Mindoro, Philippines	[62]	Iraya Mangyans possess rich traditional climate knowledge (TCK), derived mostly from nature observations. This knowledge ensures community safety, food security, and resilience to local climate change impacts.
28	The Well-Being of Adolescents in Northern Canada	[63]	The study on adolescent well-being in Canada’s Territorial North reveals substantial disparities, with many indicators notably worse specifically for Aboriginal youth, including factors such as income, household education, food insecurity, obesity and overweight, and oral and mental health.
29	Mothers’ groups enrich diet and culture through promoting traditional Quichua foods	[27]	In Ecuador, a mothers’ club nutritional intervention successfully integrated wild greens into children’s diets, enhancing food security and nutritional value, as well as self-efficacy and cultural identity for Quichua women.
30	A guiding framework for needs assessment evaluations to embed digital platforms in partnership with Indigenous communities	[64]	In community-based research, digital platforms enhance engagement with Indigenous communities. Key priorities requiring digital initiatives like the coronavirus, climate change, mental health, and food security were identified. Recommendations highlight self-governance and data sovereignty in implementing digital infrastructure for needs assessments.
31	School of Living Traditions on Aeta Magbukon Indigenous knowledge: promoting Indigenous food plants for food security	[65]	The study promotes Indigenous food plants (IFP) to assess food insecurity through a school of living traditions (LT) on the Indigenous knowledge (IK). Emphasizes SLTs’ role in preserving cultural heritage and highlights the existing gap in investigation the nutritional value and health benefits of IFPs.
32	Empowering Middle-Aged Women to Bolster Food Security in their Communities	[66]	The Zero Hunger College in the Philippines tackles malnutrition and lack of community outreach challenges. Empowering women to combine Indigenous knowledge and modern technology in agriculture, aiming to uplift communities for greater food security and economic stability.
33	Exploring oral health and hygiene practices in the Algonquin community of Rapid Lake, Quebec	[67]	The study explores oral hygiene practices in the Algonquin community, highlighting the relevance of implementing systemic interventions to promote children’s oral health, addressing social factors like cultural traditions, economic and food security, and housing status.
34	Foods from the wild: Local knowledge, use pattern and distribution in Western Nepal	[68]	The study in the Annapurna Conservation Area reveals wild edible plant species are crucial for the Gurung community’s food security and income. However, a gradually decreasing availability is perceived, so community engagement in their conservation is suggested.
35	Household food insecurity, dental caries, and oral-health-related quality of life in Brazilian indigenous adults	[69]	The study in Guarita Indigenous Land in Brazil associates food insecurity with oral health life quality perception and social determinants of health, revealing high social vulnerability.
36	Evolution of social food assistance programs in Mexico through Ensanut MC 2016 data	[28]	Study on Mexico’s Social Food Assistance Programs (SFAP) finds coverage targets mostly Indigenous and low-socioeconomic households facing food insecurity, revealing the necessity to review and redirect resources to the greater nutritional vulnerability population.
37	Household Food Insecurity and the Nutritional Status of Children Aged 6–59 Months: Insights from Rural Indigenous Garo Tribes of Meghalaya, India	[70]	Child malnutrition is a pressing issue among Meghalaya’s Garo tribe. A study on household food security emphasizes enhancing access to better child nutrition, suggesting local food system revitalization for self-sufficiency.
38	Social determinants and lifestyle risk factors only partially explain the higher prevalence of food insecurity among Aboriginal and Torres Strait Islanders in the Australian state of Victoria: a cross-sectional study	[71]	This study explains the relation between food insecurity and Aboriginal/Torres Strait Islander status in Victoria. Although food insecurity is higher among Aboriginal/Torres Strait Islanders than their non-Indigenous counterparts, it was only partially explained by social determinants and lifestyle risk factors. The disparity remains, calling for further research and corrective action.
39	Food insecurity among older Aboriginal and Torres Strait Islanders	[72]	Prevalence and relations of food insecurity in older Aboriginal and Torres Strait Islanders, finding geography, language, and low socio-economic status were highly correlated with food insecurity, highlighting the need for policies to correct this issue.
40	Predictors of household food insecurity and relationship with obesity in First Nations communities in British Columbia, Manitoba, Alberta and Ontario	[73]	The study delves into food insecurity in Western and Central Canada’s First Nations communities and its relationship with obesity, revealing correlated factors such as social assistance, low education, and specific demographics. The findings underscore the need for Indigenous-led, culturally appropriate approaches to address food security and nutrition for holistic wellness and chronic disease prevention.
41	Food insecurity and social inequalities in households headed by older people in Brazil: a secondary cross-sectional analysis of a national survey	[29]	Food insecurity in older-headed households correlates with social inequalities in Brazil, particularly affecting females, Indigenous people, those with little education, and low-income individuals. The study emphasizes the urgency of further research to address challenges faced by this particular group.
42	Food insecurity among Inuit preschoolers: Nunavut Inuit Child Health Survey, 2007–2008	[74]	Inuit preschoolers face widespread food insecurity, highlighting the urgent need for interventions to ensure their health and well-being.
43	From targets to ripples: tracing the process of developing a community capacity building appraisal tool with remote Australian Indigenous communities to tackle food security	[75]	A proposed model assesses remote Indigenous Australian community capacity development in food security. It emphasizes an approach considering the importance of remote Indigenous context-specific meanings and elders’ involvement to achieve sustainability.
44	Exploring and revitalizing Indigenous food networks in Saskatchewan, Canada, as a way to improve food security	[76]	The project aims to instigate the reactivation of Indigenous food networks in Saskatchewan, Canada, studying their current status. The results show strong community interest in learning about, and having better access to, traditional foods, and point out the vital role of elders in passing on traditional knowledge for addressing food insecurity.
45	Assessing risk of mercury exposure and nutritional benefits of consumption of caribou (Rangifer tarandus) in the Vuntut Gwitchin First Nation community of Old Crow, Yukon, Canada	[77]	Porcupine Caribou (principal food source for the Vuntut Gwitchin First Nation) samples are tested, being found to have a high nutritional level and representing minimal health risk from mercury exposure.
46	Indigenous Land-Based Approaches to Well-Being: The Niska (Goose) Harvesting Program in Subarctic Ontario, Canada	[78]	The Niska program aimed to revitalize goose harvesting and Indigenous knowledge among Omushkego Cree, addressing food insecurity. Qualitative measures indicated improved general well-being. Indigenous perspectives are crucial for holistic environmental and health program assessment.
47	Unsettling Food Security: The Role of Young People in Indigenous Food System Revitalization	[79]	The official assessments of Canadian food security neglect Indigenous perspectives. The connection between access to Indigenous food systems and Indigenous food security must be recognized. Indigenous children challenge settler narratives through experiential learning projects.
48	“Tinni” Rice (Oryza rufipogon Griff.) Production: An Integrated Sociocultural Agroecosystem in Eastern Uttar Pradesh of India	[80]	Traditional ecological knowledge (TEK) and cultural institutions conserve diverse crucial wild rice varieties (’tinni’) in the Bhar community. Indigenous practices, particularly the women’s role and democratic decision-making by village elders, safeguard tinni from excessive exploitation, contributing to people’s resilience and food security amid social and environmental changes.
49	A qualitative study comparing the coping strategies between food secure and food insecure households of Kaluppini Indigenous people in South Sulawesi	[81]	The study explores coping strategies for food insufficiency in Kaluppini, Indonesia. Strategies include additional income generation, dietary changes, and traditional practices, with both food-secure and insecure households reportedly making dietary changes. Encouraging home gardening is suggested for enhancing food security.
50	Impact of Euro-Canadian agrarian practices: In search of sustainable import-substitution strategies to enhance food security in subarctic Ontario, Canada	[82]	The study assesses the impact of a 20th-century agrarian settlement on Fort Albany First Nation’s food system. The initiative led to cultural disruption and soil degradation. Enhancing food security now involves agroecosystem stewardship and community-based, autonomous programs for sustainable local food production.
51	Traditions, beliefs, and indigenous technologies in connection with the edible longhorn grasshopper Ruspolia differens (Serville 1838) in Tanzania	[83]	The study explores Indigenous technologies, processing methods, and traditions related to the consumption of the longhorn grasshopper (senene) among the Haya tribe in Tanzania. Senene processing and preserving methods should be improved to promote its commercialization, thus taking advantage of its economic potential and improving incomes, livelihoods, and household food security.
52	Enacting food sovereignty in Aotearoa New Zealand and Peru: revitalizing Indigenous knowledge, food practices and ecological philosophies	[17]	The study explores Quechua and Māori Indigenous knowledge for food security. It emphasizes a food security policy framework underpinned by cultural and environmental indicators for well-being, which are aligned with food sovereignty, advocating for an Indigenous-led approach to achieve sustainable food systems.
53	Caliata: An Indigenous Community in Ecuador Offers Lessons on Food Sovereignty and Sustainable Diets	[30]	The study in Caliata, Ecuador, explores factors influencing sustainable Andean food systems. Findings emphasize agroecological practices, dietary patterns with minimal processing, and the importance of gendered agriculture. Caliata’s model offers insights into associations between healthy ecosystems, diet, rural–urban dynamics, and food security.
54	Edible wild fruit trees and shrubs and their socioeconomic significance in central Ethiopia	[84]	The ethnobotanical study in Arsi Zone, Ethiopia, investigates Indigenous knowledge and socio-economics of edible wild fruit trees and shrubs (EWFTSs). The community values them over commercial fruit, showing potential for integration into farming systems and assisting efforts to ensure food security.
55	The Center for Indigenous Innovation and Health Equity: The Osage Nation’s Mobile Market	[85]	The Osage Nation actively works to enhance tribal food sovereignty to combat chronic disease and food insecurity. Tribal farm initiatives have improved vegetable intake among Osage children, but accessibility to farm production in remote areas is still concerning. A community-based study identifies barriers, leading to a mobile market initiative to deliver healthy, tribally produced foods to areas with high food insecurity.
56	Nutrition Transition and Health Outcomes Among Indigenous Populations of Chile	[31]	Chilean Indigenous populations show similar NCD risk factors due to Western diet influences and lifestyle changes. They face elevated health risks amid market integration and dietary shifts.
